# The role of KIR genes and ligands in leukemia surveillance

**DOI:** 10.3389/fimmu.2013.00027

**Published:** 2013-02-07

**Authors:** Florian Babor, Johannes C. Fischer, Markus Uhrberg

**Affiliations:** ^1^Department of Pediatric Oncology, Hematology and Clinical Immunology, Center for Child and Adolescent Health, Heinrich Heine UniversityDüsseldorf, Germany; ^2^Institute for Transplantation Diagnostics and Cell Therapeutics, Heinrich Heine UniversityDüsseldorf, Germany

**Keywords:** NK cells, KIR, leukemia, ALL, AML, oncology, HSCT

## Abstract

The antileukemic potential of natural killer (NK) cells has been of rising interest in recent years. Interactions between inhibitory killer cell immunoglobulin-like receptors (KIR) and HLA class I ligands seem to be critically involved in the immunosurveillance process. It is also well established that mismatching of HLA class I-encoded KIR ligands in the setting of hematopoietic stem cell transplantation leads to allorecognition of leukemic cells by NK cells, which is in line with the concept of *missing-self* recognition. Recent data now suggest that *KIR* gene polymorphism constitutes another important parameter that needs to be taken into account for selection of suitable stem cell donors. Moreover, the role of *KIR* gene polymorphism for predisposition to leukemia is a current matter of debate. Here, we would like to review the role of KIR function and genetic polymorphism for recognition of leukemia and discuss the impact of these findings for developing novel concepts for NK cell-based immunotherapy strategies.

## INTRODUCTION

Natural killer (NK) cells represent a subset of innate lymphoid cells that act as first line of defense against viral infections, early cellular transformation and tumor growth (Lanier, 2005; Spits and Di Santo, 2011). The “missing-self” concept, put forward by Kärre and colleagues in the 1980s, formed the basis for understanding NK-derived allorecognition by describing one simplistic mechanism by which NK cells target tumor cells deficient in MHC-I expression (Kärre et al., 1986; Lanier, 2005). According to this hypothesis NK cells expressing a cognate inhibitory receptor for the respective major histocompatibility complex (MHC) class I molecule can detect target cells via their reduced or “missing” expression of “self” MHC class I (Ljunggren and Karre, 1990; Algarra et al., 1997). In humans this is based on human leukocyte antigen (HLA) class I-specific receptors, which are mainly encoded by the killer cell immunoglobulin-like receptor (KIR) family and the lectin-like receptor family of NKG2 genes. Additional MHC class I receptors are encoded by the LIR gene family (Colonna et al., 1997; Cosman et al., 1997). KIRs are the genetically most polymorphic of these receptors, interacting with the equally polymorphic HLA class I system. In rodents, in the absence of *KIR* genes, a comparable MHC class I-specific system is in place encoded by the Ly49 family (Karlhofer et al., 1992; Raulet et al., 1997; Trowsdale et al., 2001).

Killer cell immunoglobulin-like receptors recognize specific motifs of HLA class I molecules, which are the products of highly polymorphic genes of the MHC located on chromosome 6 (Klein and Sato, 2000a,b; Vilches and Parham, 2002; Marsh et al., 2010). HLA-C plays a key role for KIR-mediated recognition of target cells and all allelic variants invariably provide ligands for inhibitory KIR. All HLA-C allotypes carry valine (V) at position 76, while position 80 displays a dimorphism of either asparagine or lysine. HLA-C group 1 with asparagine at position 80 provides the ligand for KIR2DL2 and KIR2DL3, whereas HLA-C group 2 with lysine at position 80 provides the ligand for KIR2DL1. However, it has been shown recently that KIR2DL2 and to a lesser extend KIR2DL3 bind to certain HLA-C2 group alleles as well, while KIR2DL1 exhibits exquisite specificity for HLA-C2 only (Moesta et al., 2008). KIR3DL1 binds the HLA-B motif Bw4, which is also present on some HLA-A molecules whereas KIR3DL2 has specificity for HLA-A alleles (A3 and A11) but also for CpG oligonucleotides (Marcenaro et al., 2009). The ligand specificity of stimulatory KIR is less well described with the exception of KIR2DS1. Activating KIR2DS1 and inhibitory KIR2DL1 share ligand specificity for the HLA-C2 group, which is consistent with their highly similar extracytoplasmic domain. In case of KIR2DS4, weak but significant interactions with subsets of HLA-C alleles as well as HLA-A*11 could be detected (Graef et al., 2009). The ligands of KIR3DS1 have yet to be identified but recent studies have suggested that the HLA-Bw4-T80 allotype HLA-B*2705 is a potential putative ligand for KIR3DS1 (Martin et al., 2002; Alter et al., 2007, 2009; Korner and Altfeld, 2012). Specificity of the stimulatory KIR KIR2DS2, KIR2DS3, and KIR2DS5 is not known and might comprise also non-HLA ligands (Kim et al., 1997; Vales-Gomez et al., 1998; Winter et al., 1998; Vilches and Parham, 2002; Carr et al., 2007; Della Chiesa et al., 2008; VandenBussche et al., 2009).

Killer cell immunoglobulin-like receptors are displayed on the surface of NK cells in diverse combinations. This clonally-distributed expression mode leads to the generation of complex NK cell repertoires, which basically comprise NK cells expressing all possible combinations of receptors (Valiante et al., 1997; Raulet et al., 2001; Yawata et al., 2006; Andersson et al., 2010; Schonberg et al., 2011a). The clonal expression mode and the cell type specificity of *KIR* genes are epigenetically regulated on the levels of DNA methylation and histone modifications in addition to promoter-derived transcriptional regulation (Uhrberg, 2005b).

## NK CELL-MEDIATED TUMOR SURVEILLANCE

Natural killer cell function is determined by the net effect of signaling through several receptor families including activating, inhibiting, adhesion, and cytokine receptors. In this way, NK cells have demonstrated not only the ability of killing virally infected cells, but also of exerting antitumor cytotoxicity against lymphoblastic or myeloid hematologic malignancies and solid tumors like ovarian, breast, and colon cancer (Pende et al., 2005; Re et al., 2006; Stein et al., 2006). Notably, it has been shown that cytotoxicity of NK cells in the peripheral blood of leukemia patients is significantly reduced (Costello et al., 2002; Fauriat et al., 2007). Various mechanisms and characteristics could account for this reduced cytotoxicity: (a) an increased expression of MHC class I on leukemic blasts, (b) a reduced expression of ligands of various stimulatory NK cell receptors like NKG2D, NCR, and KIR on leukemic blasts, and (c) a reduced expression of these activating receptors on NK cells of patients with leukemia (Nowbakht et al., 2005; Szczepanski et al., 2010). Most of the investigations so far concerning the role of NK cells in leukemia dealt with adult patients suffering from acute myeloid leukemia (AML; Moretta et al., 2011). Little is known regarding the role of NK cells in the emergence and prevention of acute lymphoblastic leukemia (ALL), especially in the pediatric setting. Several studies suggest a less efficient interaction between NK cells and ALL blasts compared to the interaction between NK cells and AML blasts at the time of diagnosis (Nasrallah and Miale, 1983; Ruggeri et al., 2008). In this regard, surface density of HLA class I molecules seems to be higher on ALL compared to AML blasts (Pende et al., 2005). Other mechanisms of immune escape in ALL might be the absence or down-regulation of ligands for activating NK cell receptors and the expression of ligands for inhibitory receptors. Remarkably, information on the expression of KIR on NK cells of patients suffering from acute leukemia is scarce. Thus, the influence of an emerging acute or chronic leukemia on the functional NK cell repertoire as well as the question of adaptation to self-HLA class I and the licensing status in ALL patients remain to be analyzed in detail.

In HLA class I-matched hematopoietic stem cell transplantation (HSCT), NK cells were shown to contribute to leukemia surveillance based on the specific make-up of their KIR locus (Cooley et al., 2009, 2010). Interestingly, stimulatory *KIR* genes are emerging as important variables in this process (Venstrom et al., 2012). Since specificity of most of the stimulatory *KIR* genes is not well defined, the precise mechanism of their contribution constitutes a challenging but important question.

## LICENSING AND ALLOREACTIVITY

Since KIR and HLA class I genes segregate on different chromosomes, a tolerance mechanism has to be in place to prohibit development of autoreactive NK cells, e.g., NK cells with an inhibitory KIR for HLA class I ligands not present in the genome. NK cells expressing orphan inhibitory KIR are indeed present in most NK cell repertoires and pose a potential threat (Anfossi et al., 2006). However, only NK cells expressing inhibitory receptors for self-HLA class I acquire full functional competence, whereas potential autoreactive NK cells remain in a hyporesponsive state, a process referred to as education or licensing (Fernandez et al., 2005; Kim et al., 2005, 2008; Anfossi et al., 2006; Yu et al., 2007; Yawata et al., 2008).

In clinical stem cell transplantation, licensed NK cells become potentially alloreactive when transferred in an HLA class I mismatched patient. Moreover, non-licensed NK cells might be reactivated in the cytokine-rich environment of the reconstituting hematopoietic system, providing an additional source of alloreactive NK cells (Hsu et al., 2005, 2006; Clausen et al., 2007; Miller et al., 2007). In this regard, it could be shown that NK cells can be aberrantly activated after transplantation and achieve effector function and functional competence in spite of lacking class I ligand for donor inhibitory KIR (Hsu et al., 2005, 2006; Clausen et al., 2007; Miller et al., 2007; Yu et al., 2009). Moreover, Yu et al. (2009) revealed that in HSCT settings “unlicensed” NK cells with inhibitory KIR for non-self-HLA are functional while “licensed” NK cells with inhibitory KIR for self-HLA appeared to be hyperresponsive. These findings were opposed by Bjorklund et al. (2010) who demonstrated that NK cells without self-HLA receptors remained hyporesponsive after HSCT thus remaining tolerant. In contrast, Vago et al. (2008) showed a general hyporesponsiveness of NK cells in the presence of leukemic blasts. Licensed and unlicensed NK cell subsets with alloreactive potential can be qualitatively predicted in the HSCT setting as outlined in **Table 1** and the size of the alloreactive populations estimated by flow cytometry.

**Table 1:** Pattern of NK cell licensing and alloreactivity for HLA-C-encoded KIR ligands.

Donor	Education		Recipient	Alloreactive NK cells	
KIR ligand	Licensed	Unlicensed	KIR ligand	Licensed	Unlicensed
C1/C1	KIR2DL3	KIR2DL1	C1/C1	–	KIR2DL1
C1/C2	KIR2DL1/KIR2DL3	–	C1/C1	KIR2DL1	–
C2/C2	KIR2DL1	KIR2DL3	C1/C1	KIR2DL1	–
C1/C1	KIR2DL3	KIR2DL1	C1/C2	–	–
C1/C2	KIR2DL1/KIR2DL3	–	C1/C2	–	–
C2/C2	KIR2DL1	KIR2DL3	C1/C2	–	–
C1/C1	KIR2DL3	KIR2DL1	C2/C2	KIR2DL3	–
C1/C2	KIR2DL1/KIR2DL3	–	C2/C2	KIR2DL3	–
C2/C2	KIR2DL1	KIR2DL3	C2/C2	–	KIR2DL3

*Of note, to keep the scheme simple the contribution of KIR2DL2 and KIR2DS1 were not considered here*

Another interesting facet of KIR-mediated alloreactivity is given by the recently described education of stimulatory KIR. It was shown that NK cells expressing KIR2DS1, which is an activating receptor specific for the HLA-C2 allotype are functional only when derived from HLA-C1/C2 or HLA-C1/C1 donors but hyporesponsive in donors homozygous for HLA-C2. The data imply that expression of KIR2DS1 in the presence of HLA-C2 generally renders NK cells hyporesponsive independent of inhibitory KIR or CD94/NKG2A expression (Fauriat et al., 2010). In line with this, in a recent evaluation of allogeneic HSCT for AML, grafts possessing KIR2DS1 on a C1/C1 background were associated with the lowest incidence of relapse (Venstrom et al., 2012). However, no significant effect of KIR2DS1 was seen in C2/C2 patients, which generally exhibited worse outcome compared to C1/C1. It was thus suggested that the lack of C2/C2 (or alternatively the presence of C1/C1) is another dominant genetic influence in AML patients that is independent of KIR2DS1 (Fischer et al., 2007; Fischer and Uhrberg, 2012). Remarkably, Pende et al. (2009) reported that KIR2DS1-expressing NK cell clones generated from C2/C2 recipients following haploidentical transplantation were highly cytolytic against patients’ C2/C2 leukemic blasts and overriding inhibition by NKG2A or inhibitory KIR. Clearly, the clinical role of KIR2DS1 and other stimulatory KIR for NK cell alloreactivity should be clarified in further studies.

## DEVELOPMENT OF ALLOGENEIC NK CELL REPERTOIRES

Natural killer cells develop from hematopoietic progenitor cells (HPC) in distinct stages, defined by a panel of surface markers (Yokoyama et al., 2004; Freud et al., 2006; Caligiuri, 2008). NK progenitor cells (CD34^dim^/CD117^-^/NKG2A^-^) differentiate into immature CD56^+^ NK cells, which still lack the CD94/NKG2A heterodimer (CD34^-^/CD117^+^/CD56^+/-^). In a next step, these immature NK cells develop into CD56^bright^ NK cells, which express CD94/NKG2A (CD34^-^/CD117^+/-^/CD94^+^). Finally, mature NK cells are characterized by CD56^dim^, CD16^+^ and either NKG2A or KIR expression (CD34^-^/CD117^-^/CD94^+^/CD16^+^/KIR^+^; Freud et al., 2006). In this context, it was already shown that CD56^bright^ cells found in peripheral blood and secondary lymphoid organs can be directly differentiated into mature CD56^dim^CD16^+^ NK cells (Ferlazzo et al., 2004; Chan et al., 2007; Romagnani et al., 2007; Huntington et al., 2009).

After HSCT, NK cells are known to be the lymphocyte subset showing the fastest reconstitution *in vivo*, which is a prerequisite to efficiently exert antileukemic effects early after HSCT (Pfeiffer et al., 2010). They are CD56^+^ and predominantly CD16^-^ and KIR^-^. It could be shown that CD56^dim^ NK cells from patients who recently underwent hematopoietic transplantation transiently overexpress NKG2A, compared with cells from healthy donors (Dulphy et al., 2008; Vago et al., 2008; Beziat et al., 2009, 2010). In many respects, the early recovering NK cells closely resemble the CD56^bright^ subset of peripheral blood NK cells, whereas the fraction of cells corresponding to the CD56^dim^CD16^+^ NK cell fraction increases and predominates at later times after transplantation (Shilling et al., 2003). This orderly appearance of CD56^bright^ and CD56^dim^CD16^+^ subsets supports the model that CD56^bright^ cells are the principal progenitors of mature CD56^dim^CD16^+^ NK cells.

**FIGURE 1:**
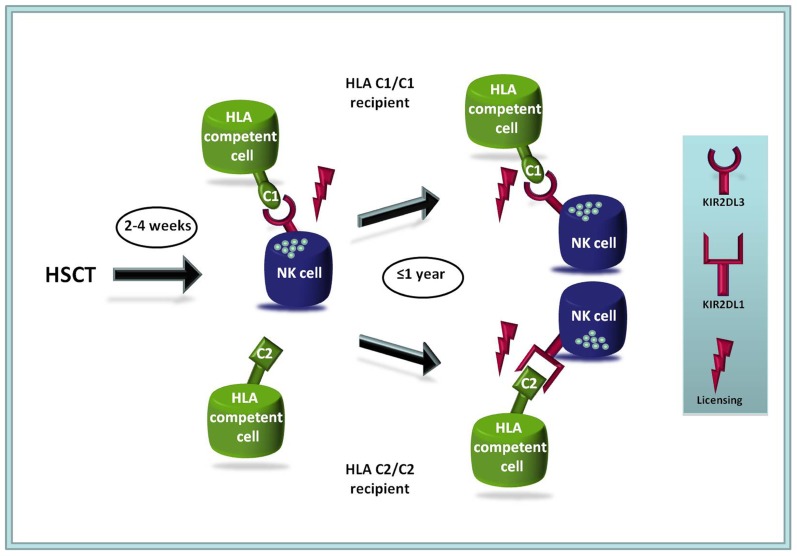
**Model of NK cell reconstitution and NK cell receptor expression following allogeneic HSCT**. The HLA-C1-specific KIR2DL2/3 receptor is expressed at earlier time points on reconstituting NK cells than the HLA-C2-specific KIR2DL1 (Shilling et al., 2003; Nguyen et al., 2005; Fischer et al., 2007). Thus, in the clinical setting C2/C2 recipients possess a lower frequency of functionally competent NK cells (expressing KIR2DL1) than C1/C1 patients (expressing KIR2DL2/3), which may lead to a disadvantage in the early phase following HSCT. The model is compatible with decreased overall survival rates and increased relapse rates in C2/C2 individuals that were found in several different studies (Fischer et al., 2007, 2012; Venstrom et al., 2012).

Another factor that influences the composition of allogeneic NK cell repertoires following HSCT is the sequential mode of KIR acquisition during NK cell differentiation. *In vitro*, NK cell differentiation from hematopoietic progenitors leads to early expression of KIR2DL2/3 whereas KIR2DL1 is only expressed at later time points (Miller and McCullar, 2001; Schonberg et al., 2011b). The model of differentiation is given in **Figure 1**. Similar observations were made in studies following the reconstitution of NK cell repertoires following HSCT (Shilling et al., 2003). These patterns of expression influence the overall frequency of functionally competent NK cells including putative leukemia-reactive clones. In this context, it could be shown that the presence of HLA-C1, which is the ligand for the early-expressed KIR receptor KIR2DL3 was associated with increased survival if present in the recipient, whereas the presence of HLA-C2 ligands in the recipient constituted a risk factor (Fischer et al., 2007). These findings were in line with previous clinical observations (Giebel et al., 2003; Hsu et al., 2005). Notably, HLA-C allele matching (four-digit level) had differential effects in HLA-C1 (*C1/C1*) and HLA-C2 (*C1/C2* and C2/C2) subgroups of patients: in HLA-C1 patients with myeloid leukemia, HLA-C-matching was associated with improved survival as described previously (Morishima et al., 2002; Flomenberg et al., 2004; Petersdorf et al., 2004; Woolfrey et al., 2011). Surprisingly, the opposite was true for HLA-C2 patients, which profited from having an HLA-C allele mismatch on the allelic level (Fischer et al., 2012), i.e., two different HLA-C-alleles that both belong to the HLA-C2 ligand group. HLA-C2 patients with these allele-mismatched, but “KIR-neutral” donors generally showed superior survival with substantial improvement in treatment related mortality and relapse incidence compared to HLA-C1 patients with HLA-C mismatches. The data suggest that HLA-C mismatching should be avoided in HLA-C1 patients as reported previously but might be favorable for HLA-C2 patients. Furthermore, the study suggested a novel model in which matched HLA-C1 and mismatched HLA-C2 patients constitute a low-risk group, whereas mismatched HLA-C1 and matched HLA-C2 patients would represent a high-risk group with inferior overall survival, increased TRM and higher relapse rate [in myeloid leukemia and myelodysplasia syndrome (MDS); Fischer et al., 2012].

## KIR GENETICS AND ALLOREACTIVITY

Two *KIR* haplotype groups, *A* and *B*, have been identified based on differential *KIR* gene content (Uhrberg et al., 1997). Group *A* and *B* haplotypes have four framework genes in common: the *KIR* gene cluster is flanked by the non-expressed *KIR3DL3* at the centromeric end, *KIR3DL2* at the telomeric end, with* KIR3DP1* and *KIR2DL4* in the middle. Deletion and duplication of genes have led to many different haplotypes (Vierra-Green et al., 2012). Group *A* haplotypes are present in all populations and consist of five productive inhibitory *KIR* (*KIR2DL3*, *KIR2DL1*, *KIR2DL4*, *KIR3DL1*, *KIR3DL2*), and the stimulatory *KIR2DS4*, which is frequently present in an allelic variant that is not expressed on the cell surface (Maxwell et al., 2002). Group *B* comprises *KIR* haplotypes with diverse gene content including several genes (*KIR2DL2*, *KIR2DL5*, *KIR2DS1*, *KIR2DS2*, *KIR2DS3*, *KIR2DS5*, and *KIR3DS1*) that are not part of group *A* haplotypes. As a consequence, most group *B* haplotypes encode more activating KIRs compared to group *A* haplotypes. All individuals can be categorized as having one of two KIR genotypes: *A/A*, which is homozygous for group *A* KIR haplotypes, or *B/x*, which contains either one (*A/B* heterozygotes) or two (*B/B* homozygotes) group *B* haplotypes.

There have only been a few studies regarding the impact of genetic polymorphisms of activating and inhibitory KIR in humans on the susceptibility and resistance to leukemia. Verheyden et al. (2004) revealed an association of the inhibitory *KIR2DL2* gene with leukemia. In a polymorphic cohort of AML, ALL, CML (chronic myeloid leukemia), and CLL (chronic lymphoblastic leukemia) patients they found a significantly increased frequency of the KIR haplotype combination *A/B* in leukemic patients compared to controls. This finding was related to the high prevalence of the inhibitory *KIR2DL2* gene. Another significant association with leukemia was found for two distinct KIR genotypes, which included all inhibitory KIRs (Uhrberg et al., 2002; Verheyden and Demanet, 2008). In a more recent study of pediatric ALL patients, Almalte et al. (2011) reported a highly significant association between the presence of stimulatory *KIR* genes and decreased risk for ALL. The risk for ALL progressively decreased with the cumulative number of stimulatory *KIR*. In a subsequent study, no significant association was found between childhood ALL and stimulatory *KIR* genes (Babor et al., 2012). In the latter study neither stimulatory nor inhibitory genes exhibited a significant deviation in frequency from an ethnically matched control group. Analyses of the cumulative number of stimulatory and inhibitory *KIR* genes as well as further analysis of telomeric and centromeric *KIR* haplotypes did not reveal any significant difference to the control cohort. Similarly, in a recent study analyzing a large cohort of AML patients, frequencies of stimulatory *KIR* genes were again not different to published healthy donor cohorts (Venstrom et al., 2012). More thorough analysis of *KIR* genes on the allelic level will be necessary to settle the question if *KIR* genes constitute a major disease susceptibility locus for acute leukemia in children and adults. Notably, the identification of single *KIR* genes as disease markers is generally complicated by the fact that groups of *KIR* genes are tightly linked in a small number of common centromeric and telomeric haplotypes (Uhrberg, 2005a). Moreover, centromeric KIR genes could be linked to the neighboring LIR gene family including inhibitory and stimulatory LIR genes that are known to be expressed on NK cells as well (Cosman et al., 1997; Young et al., 2001).

In the clinical transplant setting, Stringaris et al. (2010) revealed that three donor *B* haplotype *KIR* genes, *KIR2DL5A*, *KIR2DS1*, and *KIR3DS1*, were associated with significantly less relapse in patients with AML undergoing HLA-identical sibling HSCT. The authors could not confirm these findings in patients with other myeloid or lymphoid malignancies. Recent data suggest evidence of beneficial effects of KIR *B* genotype donors in AML patients in an unrelated HLA-matched HSCT setting (Cooley et al., 2009, 2010). Cooley et al. (2009) analyzed the effect of KIR genotypes on outcome in a comparatively homogeneous study cohort of 448 AML patients who received T cell replete transplants from unrelated donors. They could show that the use of donors with KIR group *B* haplotypes was associated with significant improvement in relapse-free and overall survival, with 30% better relative risk in both endpoints. The same group subsequently performed analyses regarding gene content motifs, which further revealed that compared with group *A* haplotype motifs, centromeric and telomeric group *B* motifs both contributed to relapse protection and improved survival. Centromeric B homozygosity (*Cen-B/B*) had the strongest independent effect. Overall, significantly reduced relapse was achieved with donors having two or more *B* gene-content motifs in both HLA-matched and mismatched transplants (Cooley et al., 2010). In contrast, Venstrom et al. (2012) revealed in their study of AML transplant patients a beneficial effect of telomeric but not centromeric genes, namely *KIR2DS1* and *KIR3DS1*. Together, these findings highlight the need for further studies of KIR polymorphism in HSCT, possibly on the allelic level, to enable incorporation of *KIR* gene content status along with HLA-matching in future algorithms for donor selection in allogeneic/haploidentical HSCT settings.

## EXPLOITING KIR AND KIR LIGAND MISMATCHING IN HSCT

In HSCT settings, donor-derived T cells are essential not only for early cellular engraftment following HSCT but also for eradication of remaining leukemic blasts. On the other hand, T cells are major effectors of rejection or acute or chronic graft versus host disease (GvHD). T cell-mediated alloreactivity is directed against histocompatibility antigens not only displayed on the stem cell recipient’s leukemic cells but also on other tissues. Especially cytotoxic CD8^+^ effector T cells may then attack tissues such as liver, intestines, and skin, which results in acute or chronic GvHD. Currently, in order to avoid GvHD various manipulations of HSCT grafts such as CD6^+^ T cell depletion (Soiffer et al., 2001), CD3^+^/CD19^+^ T and B cell depletion (Federmann et al., 2012) or α/β T cell (Handgretinger, 2012) depletion are implemented in different transplant centers. With this approach, the numbers of potentially graft rejecting or GvHD causing T cells can be reduced considerably while HPC and NK cells remain in the graft and can be transferred into the patient. Such grafts, mainly consisting of mature NK cells, CD34^+^ stem cells and only a few remaining T cells (max. 5 × 10^4^/kg BW) of donor origin repopulate the recipient’s hematopoietic system and rebuild the recipient’s new immune system that quickly reconstitutes immunity to viral and bacterial infections and eliminates leukemic blasts that survived chemotherapy (graft versus leukemia effect – GvL). Especially in CD3^+^/CD19^+^ depleted grafts large numbers of mature donor NK cells are co-transfused.

Previous *in vitro* studies already demonstrated the alloreactive potential of NK cells (Ciccone et al., 1992; Colonna et al., 1992) and showed that the kind and frequency of alloreactive NK cells can be predicted if KIR expression in the donor and presence of KIR ligands in the recipient are known (Valiante et al., 1997). The clinical contribution of NK cells to alloreactivity was impressively shown by Ruggeri et al. (2002) who demonstrated a strong GvL effect for AML patients receiving a haploidentical allogeneic transplant mismatched for HLA-C KIR ligands. In this setting alloreactive donor-derived NK cells are thought to promote engraftment, reduce GVHD, and decrease leukemic relapse. Whereas some subsequent studies did confirm the KIR ligand mismatch model (Giebel et al., 2003) others could not find a beneficial effect of KIR ligand mismatching (Davies et al., 2002; Hsu et al., 2006; Miller et al., 2007), which might be explained by specific differences in HSCT protocols performed in the different transplant centers. Subsequently, several modified donor selection models were suggested based on the consideration that donor NK cells expressing a certain inhibitory KIR would be alloreactive if the respective ligands (C1, C2, or Bw4) in the patient are missing. This model was originally proposed by Leung et al. (2004) and demonstrated better clinical outcome in haploidentical HSCT for pediatric lymphoid leukemia thereby proofing that NK cell-mediated relapse control is not restricted to myeloid leukemia. Hsu et al. (2005) applied the same model to HLA-identical HSCT showing that patients with missing KIR ligands for which the donor possessed a specific KIR exhibited increased survival through decreased relapse incidence in AML and MDS patients. An overview of studies investigating the role of *KIR* genes in clinical stem cell transplantation is given in **Table 2**.

**Table 2:** An overview of studies analyzing the role of KIR for susceptibility and clinical HSCT for leukemia.

	KIR/model	Observation	Disease	Treatment	Reference
Genetic associations	*KIR2DL2*	Increased frequency	AML/ALL	–	(Verheyden et al., 2004)
	*KIR-S*	Decreased frequency	ped. ALL	–	(Almalte et al., 2011)
	*KIR-L / KIR-S*	No association	ped ALL	–	(Babor et al., 2012)
Inhibitory KIR	Missing KIR ligand	Relapse ↓	ped. AML/ALL	Haploidentical	(Leung et al., 2004)
	Missing KIR Ligand	Survival ↑, Relapse ↓	AML/ALL	MSD HSCT	(Hsu et al., 2005)
	KIR ligand mismatch	Relapse ↓, Survival ↑	AML	Haploidentical	(Ruggeri et al., 2007)
	KIR3DL1	Survival ↓	Acute leukemia	Unrelated HSCT	(Gagne et al., 2009)
Stimulatory KIR	KIR2DS2	Survival ↓	AML (ALL)	MSD HSCT	(Cook et al., 2004)
	KIR3DS1	Survival ↓	Acute leukemia	Unrelated HSCT	(Gagne et al., 2009)
	KIR2DS1	Relapse ↓	AML	Unrelated HSCT	(Venstrom et al., 2012)
Haplotype structure	Group B	Relapse ↓ chronic GvHD ↑	Adult AML	Unrelated HSCT	(Cooley et al., 2009)
	Group B haplotype score	Relapse ↓	AML (ALL)	Unrelated HSCT	(Cooley et al., 2010)

*HSCT, hematopoietic stem cell transplantation; AML, acute myeloid leukemia; ALL, acute lymphoblastic leukemia; Cen, centromeric; MSD, matched sibling donor; ped, pediatric; indiv, individuals.*

## NK CELL IMMUNOTHERAPY: ONLY AT THE BEGINNING OF GREAT POSSIBILITIES?

Cytokines such as IL-2, IL-15, and IL-21, as well as the combination of IL-12 and IL-18 are known to activate NK cells (Carson et al., 1995; Singh et al., 2000; Trinchieri, 2003; Young and Ortaldo, 2006). Coadministration of such NK cell-activating cytokines have the potential to increase survival of adoptively transferred NK cells, enhance proliferation, up-regulation of cytotoxic and adhesion molecules, augment cytokine production and increase GvL effects (Becknell and Caligiuri, 2005). IL-2 activated NK cells kill tumor cells *in vitro* and *in vivo* by localizing to the tumor sites to elicit their cytotoxic action and they prolong the survival of tumor bearing mice. This was seen in mice with multiple myeloma (Alici et al., 2007) and in NOD/SCID mice with metastatic neuroblastoma (Castriconi et al., 2004). Unfortunately, administered cytokines such as IL-2, IL-15, or IL-18 are rapidly cleared, thus requiring repeated injections (subcutaneous) of large amounts. This in turn increases the potential for toxicity considerably and furthermore might elicit activation of unwanted cell populations or even cause activation-induced cell death of NK cells. IL-2 but not IL-15 is known to be involved in the maintenance of T regulatory cells (Treg), which could impair antitumor immune response (Waldmann, 2006). Thus, IL-15 administration holds promise to specifically boost NK cell alloreactivity without undesired stimulation of Tregs. Importantly, the use of IL-15 as GMP product has recently been approved by the U.S. Food and Drug Administration (FDA). However, since full biological activity of IL-15 is dependent on its trans-presentation on the cell surface, suitable application strategies have to be developed to increase biological stability and activity. These include administration of pre-associated complexes of IL-15 with soluble IL-15Rα-IgG1-Fc or stimulation of native IL-15Rα expression with, e.g., anti-CD40 (Zhang et al., 2009; Steel et al., 2012).

Advanced cell therapy trials with donor NK cells post-haploidentical stem cell transplantation provide a promising treatment option for patients with high risk leukemia and tumors (Leung et al., 2004, 2005). While the established adoptive T cell therapies are associated with the risk of GvHD, NK cells mediate GvL effects without substantial induction of acute GvHD. These findings encouraged scientists and clinicians to conduct clinical trials utilizing adoptive transfer of cytokine-activated NK cells, which could serve as an attractive cell therapy option not only in recipients of haploidentical stem cell transplantation but in other oncologic diseases as well (Miller et al., 2005; Passweg et al., 2006; Rubnitz et al., 2010). In 2005, Miller and colleagues were the first to infuse haploidentical NK cells into AML recipients in a non-transplant setting (Miller et al., 2005). Before NK cell infusion patients received an intensive immunosuppressive regimen of low-dose cyclophosphamide and methylprednisolone or fludarabine. Successful transfer of haploidentical KIR ligand-mismatched NK cells was followed by a subcutaneous administration of IL-2 for 2 weeks. NK cells expanded *in vivo* and were functional at day 14 following infusion (Miller et al., 2005). But still, the intensive conditioning regimen and high doses of IL-2 resulted in significant hematologic and non-hematologic toxicities as well as prolonged hospitalization. In order to further optimize this strategy of donor-derived NK cell transfer, Rubnitz et al. (2010) extended investigations concerning the safety and feasibility of low-dose immunosuppression followed by NK cell infusion. This study on transfer of alloreactive haploidentical KIR ligand-mismatched NK cells has been performed in children with AML after achievement of complete remission. In order to minimize toxicity while still allowing engraftment of haploidentical NK cells a low-intensity regimen was administered to patients followed by NK cell infusion and multiple doses of IL-2. The study gave encouraging results, and has allowed commencement of a phase II trial as consolidation therapy in children with AML. In multiple myeloma, infusion of haploidentical KIR ligand-mismatched NK cells after conditioning therapy with melphalan and fludarabine has been proposed in relapsed patients (Shi et al., 2008). Curti et al. (2011) demonstrated that infusion of purified NK cells is feasible and safe in elderly patients with high-risk AML and these donor alloreactive NK cells were shown to kill recipient leukemia cells. Again, no NK cell-related toxicity, including GVHD, was observed. Donor-versus-recipient alloreactive NK cells were shown *in vivo* by the detection of donor-derived NK clones that killed recipient’s targets.

Together, these trials show that adoptive immunotherapy with haploidentical NK cells obtained from KIR ligand-mismatched donors is feasible and safe in adults as well as in children. Nevertheless, further studies are highly warranted to specifically assess the role of NK cell therapy to elucidate which patient collective (AML/ALL/adults/children) is best suited at which stage of disease (remission/relapse) and at which stage of therapy (after/prior to/without HSCT). Furthermore, these studies should address the question, which NK cell subset (single KIR^+^ NK cells, licensed or unlicensed) mediates strongest reactivity against leukemia.

## CONCLUSION

*KIR* genes have two unusual features that could be harnessed for improving current HSCT strategies: firstly, *KIR* genes are highly polymorphic leading to novel opportunities beyond classical HLA matching strategies for selection of optimal stem cell donors based on *KIR* genetic diversity. Secondly, KIR are expressed in a clonally distributed manner, which leads to the formation of NK cell repertoires encompassing tolerant as well as potentially alloreactive NK cell clones. Both features have a great potential to be exploited in the setting of clinical stem cell transplantation.

While recent studies could not detect a predisposition for leukemia based on presence or absence of particular inhibitory and stimulatory *KIR* genes or KIR haplotype classification, there is growing evidence for beneficial effects of KIR B genotype donors in HSCT for AML. In general, NK cells and their variable capability of killing residual leukemic blasts become a criterion of growing importance as clinicians are offered another important factor concerning donor selection in unrelated, sibling, and haploidentical transplant settings along with HLA matching, CMV status, blood group, age, and gender. Furthermore, recent investigations suggest that homozygous HLA-C2 stem cell transplant recipients constitute a high-risk group that needs special attention. In this regard, recent data suggest that survival of C2/C2 patients might be improved by allele mismatching of donor/recipient pairs for HLA-C2, a novel concept that has to be confirmed in retrospective studies of larger cohorts. On the basis of optimally defined matching/mismatching strategies an important next step for successful NK cell-based intervention in HSCT will be to qualitatively and quantitatively define patient-specific alloreactive subsets among donor NK cell repertoires opening the possibility to stimulate, enrich, and expand those subsets *in vitro* or *in vivo* for immunotherapy.

Antileukemic NK cells, either allogeneic or unlicensed autologous NK cells, emerge as a feasible therapy option and might improve clinical outcome in myloid leukemia. Further integration into established HSCT protocols may indeed improve survival rates in adults and children. It will be an important task to define, which patients are most suitable for NK cell-mediated antileukemic therapy based on incorporation of multiple factors like genetic predisposition, HLA, KIR and the nature of leukemic disease. It would be desirable to expand the possible range of applications to ALL and lymphoma as well as toward NK cell-based therapy of solid tumors. Toward this goal, our biological understanding of NK cell function and interaction with leukemic target cells has to be further improved.

## Conflict of Interest Statement

The authors declare that the research was conducted in the absence of any commercial or financial relationships that could be construed as a potential conflict of interest.
